# Gliosarcome: tumeur rare du système nerveux central - présentation de deux cas

**DOI:** 10.11604/pamj.2015.22.305.7852

**Published:** 2015-11-25

**Authors:** Zenab Alami, Touria Bouhafa, Fatimazahra Farhane, Abderahmane Elmazghi, Khalid Hassouni

**Affiliations:** 1 Service de Radiothérapie-Curiethérapie, CHU Hasssan II, Fes, Maroc

**Keywords:** Gliosarcome, chirurgie, radiothérapie, chimiothérapie, système neveux central, gliosarcoma, surgery, radiotherapy, chimiotherapy, central nervous system

## Abstract

Le gliosarcome est une tumeur primitive mixte du système nerveux central, caractérisée par une prolifération biphasique associant un contingent glial de type glioblastome et un contingent sarcomateux. L’âge moyen de survenue de cette tumeur varie de 40 à 60 ans, avec un sex-ratio homme/femme de 1,8/1. Nous rapportons ici deux cas de gliosarcome traités dans notre service Le traitement standard consiste en une résection chirurgicale de la tumeur suivie d'une radiothérapie externe et parfois d'une chimiothérapie.

## Introduction

Le gliosarcome est une variante du glioblastome, caractérisée par une prolifération biphasique du système nerveux central associant un contingent glial de type glioblastome et un contingent sarcomateux. Selon l'organisation mondiale de la santé (OMS), cette tumeur représente 2% des glioblastomes, et est classée de grade IV. Ce sont des tumeurs rares, représentant 0,8 à 8% de l'ensemble des glioblastomes. L’âge moyen de survenue de cette tumeur varie de 40 à 60 ans, avec une légère prédominance masculine (sex-ratio est de 1,8). Le traitement consiste en une exérèse chirurgicale suivie par une radiothérapie externe et une chimiothérapie dans certains cas. Nous rapportons deux cas de glioblastome traités dans notre département.

## Patient et observation

### Observation n°1

**Figure 1:**
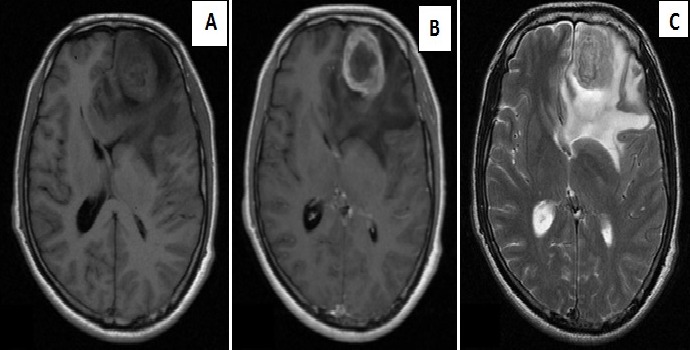
IRM cérébrale en coupes axiales séquences pondérées T1 sans (A) et avec gadolinium (B), séquence T2 (C), montrant un processus intraparenchymateux frontal gauche, en discret hyposignal T1, discret hypersignal T2, rehaussé en anneau après injection de Produit de contraste, entouré d'un important œdème cerebral

**Figure 2:**
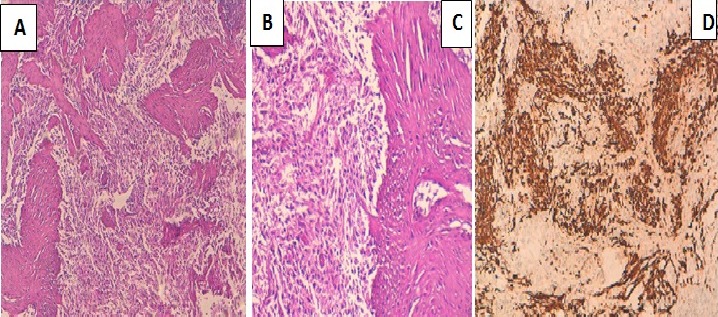
(A) prolifération tumorale à double composante gliale (B) et sarcomateuse (C) (HES×100) immunomarquage positif de la composante gliale par l'anticorps anti-GFAP (D), négatif sur la composante mésenchymateuse (HES×100)

Patient de 53 ans sans antécédents pathologiques notables qui présente 5mois avant son admission un syndrome d'HTIC d'aggravation progressive compliqué d'une hémiparésie droite et des crises convulsives. Le patient a bénéficié d'une TDM puis une IRM cérébral objectivant la présence d'un Processus lésionnel intraparenchymateux frontal gauche mesurant 45/30 mm, en hyposignal T1, discret hypersignal T2, rehaussé de façon hétérogène après injection de PDC, entouré d'un important œdème cérébral et déterminant un effet de masse sur les structures de la ligne médiane évoquant un processus tumoral glial (Figure 1). Le diagnostic d'une lésion tumorale frontale gauche a été alors retenu et le patient a bénéficié d'une exérèse chirurgicale complète de la tumeur. Les suites opératoires étaient favorables avec un début de récupération du déficit moteur. L'examen histologique a montré une prolifération tumorale maligne disposée en nappes diffuses sur fond fibrillaire; cette prolifération tumorale est faite de cellules pléomorphes de grande taille aux noyaux atypiques hyperchromatiques avec de nombreuses mitoses. Il s'y associe une composante de cellules fusiformes atypiques avec un noyau ovoïde et hyperchromatique. Des foyers de nécrose entourés par une palissade de cellules tumorales étaient également notés donnant un aspect en carte géographique (Figure 2). Le stroma tumoral comportait une prolifération endothélio- capillaire. Une étude immunohistochimique a été réalisée, les cellules tumorales de la composante gliale exprimaient la GFAP (Figure 2), la composante mésenchymateuse avait un marquage positif pour la desmine et l'AML (actine musculaire lisse). Le patient a été référé à notre service. Il a bénéficié d'une Radiothérapie adjuvante par deux champs latéraux isocentrique, une dose totale de 60Gy en 30 fractions (2Gy/fraction). Un mois après la fin de la radiothérapie, le patient a présenté de multiples tuméfactions du cuir chevelu en regard du champ d'irradiation. L’évolution clinique était marquée par une dégradation de l’état général et neurologique. Le scanner cérébrale de contrôle a montré une importante récidive tumorale frontale gauche déterminant un engagement sous falcoriel, associée à de multiples lésions sous cutanées extra-crâniennes. Le patient est décédé quatre mois après la fin de la radiothérapie.

### Observation n°2

Patiente de 51 ans sans antécédents pathologiques notables, présentant depuis quelques mois de son admission des céphalées d'aggravation progressive résistante au traitement antalgique, l’évolution été marquée par l'installation de troubles de consciences. Devant cette symptomatologie la patiente a consulté en urgence. L'examen initial a révélé une patiente avec un score de Glasgow (GCS) à 7. Un scanner cérébral a objectivé la présence d'un processus tissulaire temporal droit contenant de la nécrose et rehaussé de façon hétérogène après contraste ce processus est entouré d'un important œdème péri lésionnel en doigt de gant et mesure 56&36mm l'ensemble est responsable d'un effet de masse sur les structures de la ligne médiane avec un engagement temporal. Une IRM Cérébrale a révélé la présence du même processus expansif temporal droit mesurant 6cm de grand axe, une Infiltration en hypersignal T2 péri lésionnel, l'hippocampe est non reconnaissable au sein de la lésion effet de masse important sur le ventricule latéral avec signe d'engagement sous falcoriel effet de masse sur le mésencéphale refoulé et déformé avec signe d ‘engagement trans tentoriel descendent, effet de masse sur l'aqueduc de sylvius avec dilatation du ventricule latéral Gauche. Cet Aspect IRM est en faveur de tumeur gliale maligne temporal Dt avec effet de masse important avec signe d'engagement sous falcoriel et trans tentoriel descendant La patiente a bénéficié d'une exérèse complète de la tumeur, Les suites opératoires étaient marquées par une amélioration franche de la patiente avec un GCS à 15. A l’étude anatomopathologique: l'examen macroscopique a montré un processus temporal malin peu différencié largement nécrosé évoquant soit un glioblastome soit une métastase d'un processus carcinomateux. L’étude microscopique a montré une prolifération tumorale largement nécrosée d'architecture diffuse, la densité cellulaire est élevée. Les cellules tumorales sont de taille moyenne à cytoplasme éosinophile moyennement abondant, aux noyaux ovoïdes, anisocaryotiques, nucloéoles, montrant des figures de mitose. On a noté par endroit une différenciation sarcomatoide mise en évidence par l'immunohistochimie. La patiente a été référé à notre service, elle a bénéficié d'une radio-chimiothérapie concomitantes selon le Protocol Stupp: Radiothérapie de 60 Gy en 30 fractions et six semaines, associée à du Témozolomide à la dose de 75mg/m2/j pendant 42 jours consécutifs, puis six cycles de 150-200mg/m2/j de j1-j5 débutant tous les 28 jours. La patiente a été perdue de vue après la fin de la chimiothérapie adjuvante.

## Discussion

Le gliosarcome est une tumeur maligne primitive du système nerveux central, rare, représentant 2% de tous les glioblastomes et 0,59-0,76% de toutes les tumeurs cérébrales [1]. C'est une tumeur biphasique, associant un contingent glial de type glioblastome et un contingent sarcomateux. Cette tumeur a été décrite pour la première fois par Stroebe en 1895 [2], et a gagné une large approbation après les descriptions histologiques détaillées faites en 1955 par Feigin et Gross; ces derniers ont décrit ces tumeurs comme des glioblastomes dans lesquels la prolifération vasculaire acquière un aspect sarcomateux [3]. Elle est actuellement définie comme étant une variante morphologique du glioblastome multiforme. Il touche plus l'homme que la femme avec une atteinte préférentielle de la tranche des 40-60 ans avec un sexe ratio homme/femme de 1,8. La localisation est essentiellement supratentorielle, touchant la région temporale dans plus de 65% des cas; les régions frontale, pariétale et occipitale peuvent être atteintes. Rarement le gliosarcome occupe la fosse cérébrale postérieure et la moelle épinière [4, 5]. L'histoire clinique est le plus souvent courte, avec une durée d'une semaine à 3mois [6], une symptomatologie polymorphe en fonction de la zone d'atteinte, Néanmoins, elle reste dominée par le syndrome d'HTIC, le déficit moteur et les crises convulsives. Des cas de gliosarcome ont été découverts au stade de métastase [7, 8]. L'aspect tomodensitométrique des gliosarcomes peut simuler un glioblastome. Le gliosarcome apparaît normalement sous forme d'une masse souvent superficielle, bien circonscrite, hyperdense avec rehaussement périphérique en couronne hétérogène ou irrégulière, entourant une hypodensité centrale correspondant des plages de nécrose et un œdème péritumoral [9]. Si la composante mésenchymateuse est importante, l'aspect est celui d'une masse hyperdense prenant le contraste de façon homogène et simulant un méningiome, mais sans base d'implantation au niveau du crâne [10]. L'aspect en résonance magnétique est caractéristique puisqu'il montre une tumeur bien limitée, intra axiale, entrant en contact avec la dure-mère, avec des zones de remaniement kystique et un ‘dème vasogénique. En T2, l'intensité du signal est intermédiaire, similaire à la substance grise, mais hypo intense en comparaison avec les autres tumeurs gliales. Après injection de gadolinium, en T1, la tumeur montre un important rehaussement en anneau. Ces zones de rehaussement sont iso-intense en T2 [11]. Le diagnostic de gliosarcome doit donc être évoqué devant toute tumeur hypo-intense en T2, de siége intra-axial primitif et rentrant en contact avec la dure mère.

Le mélange des tissus gliomateux et sarcomateux confère au gliosarcome l'aspect d'un tissus biphasique. La composante gliale est essentiellement de type astrocytaire de haut grade, de type glioblastome avec un degré variable d'anaplasie [12], La composante sarcomateuse, faite d'une prolifération en faisceau de cellules fusiformes atypiques à index mitotique élevé. La distinction entre ces deux composantes est devenue facile grâce à la combinaison de l'histochimie et de l'immunohistochimie. La disposition du collagène dans la composante mésenchymateuse est bien démontrée par la coloration au trichrome de Masson. Les fibres de réticuline ne se voient qu'autour des vaisseaux au sein de la composante gliale et sont abondantes au sein de la composante conjonctive. Cette dernière exprime la vimentine et n'exprime pas la GFAP, qui au contraire, est exprimée dans la partie gliale. La démonstration claire du caractère malin du contingent mésenchymateux GFAP-négatif et importante afin de distinguer un vrai gliosarcome d'un glioblastome avec prolifération fibroblastique [13]. La pathogénie, des gliosarcomes reste obscure. Classiquement, les tumeurs biphasiques répondent à trois mécanismes (classification de Meyer): un mécanisme de collision: les néoplasmes différents convergent pour former un seul néoplasme; un mécanisme de combinaison où une seule cellule donne naissance aux deux composantes; un mécanisme de composition où il existe une transformation maligne touchant deux tissus au même moment. La théorie le plus souvent retenue est que la composante sarcomateuse semble prendre naissance à partir des vaisceaux d'un glioblastome préexistant. Au plan génétique, Les gliosarcomes présentent un profil plus proche des glioblastomes secondaires que des glioblastomes primaires. En effet Reis et al. (2000) rapportent des mutations de TP53 dans 23%, des mutations de PTEN dans 38%, des délétions de p16INK4 dans 37% mais présente rarement une amplification de l'EGFR < 8% [14]. Le Gliosarcome est traité, de manière presque identique que le glioblastome, par la chirurgie, la radiothérapie et la chimiothérapie [15]. Il s'agit de résection partielle si la tumeur est mal limitée et infiltrante. Dans les formes avec attache durale, simulant un méningiome, l'exérèse est totale. La survie globale moyenne des patients ayant reçu une Radiothérapie adjuvante est meilleurs que ceux traité par chirurgie seule (10,6 mois vs 6,2 mois) [16]. La Radiothérapie doit être démarrée dans un délai de 4 à 6 semaines après la chirurgie sous réserve que la cicatrisation du scalp soit obtenue. Le traitement par radiothérapie se fait quotidiennement cinq jours par semaines, 2Gy par séance pour un total de 60Gy. L'identification des volumes cibles est recommandée en utilisant l'IRM post opératoire avec injection du gadolinium dans une séquence T1. Plusieurs essais sont en cours pour mieux préciser le volume à irradier, notamment en utilisant les données de l'imagerie métabolique. Des études ont montré que les zones semblant les plus actives sur le plan métabolique, notamment en spectroscopie IRM, étaient des sites préférentiels de rechutes et pourraient donc être intégrées différemment dans les plans de radiothérapie (boost par radiochirurgie ou boost intégré en imagerie médicale et de radiologie thérapeutique IMRT) Dans un volume correspondant au GTV avec une petite marge de 0,5cm, le taux de récidive observé est de 78-89%. Cette zone correspond au volume cible supposé recevoir la dose la plus élevée. Au-delà d'une distance supérieure à 2,5 cm du GTV, le taux de récidive est de 3-9%. Les volumes recommandés par les essais du RTOG 98-03 et 08-25 sont: CTV1: le lit tumorale et/ou résidu tumorale +20-25mm, CTV2= le lit tumoral et/ou résidu tumoral + 5mm. Le volume cible prévisionnel est une marge additionnel de 3-5mm. Les essais récents de l'escalade de dose par radiochirurgie, implant interstitiel ou IMRT n'ont pas montré de bénéficie sur la survie [17–19]. Le Gliosarcome est chimio-résistant mais la littérature suggère que l'utilisation du Temozolamide en même temps que la radiothérapie (75mg/m^2^) par jour 1h avant la radiothérapie et les weekends puis en adjuvant 150mg/m^2^ en 5cycles (selon le protocol stupp), améliore la survie [20]. Malgré un aspect macroscopique trompeur simulant une bonne limitation, le pronostic des gliosarcome ne diffère pas des autres Glioblastomes (Keilhues et al, 2007).

## Conclusion

Le gliosarcome est une tumeur à double composante gliale et sarcomateuse. Le tableau clinique est polymorphe, les données de l'imagerie (TDM, IRM) sont évocatrice, la confirmation est histologique et immunohistochimique. Le traitement est essentiellement basé sur la chirurgie et la radiothérapie.
